# Health predicting factors in a general population over an eight-year period in subjects with and without chronic musculoskeletal pain

**DOI:** 10.1186/1477-7525-6-98

**Published:** 2008-11-11

**Authors:** Susann Arvidsson, Barbro Arvidsson, Bengt Fridlund, Stefan Bergman

**Affiliations:** 1 Research and Development Centre, Spenshult hospital for rheumatic diseases, Oskarström, Sweden; 2 School of Health Sciences & Social Work, Växjö University, Växjö, Sweden; 3 School of Social and Health Sciences, Halmstad University, Halmstad, Sweden; 4 Gjøvik University College, Faculty of Nursing Science, Gjøvik, Norway; 5 School of Health Sciences, Jönköping University, Jönköping, Sweden

## Abstract

**Background:**

Many factors are proposed to be associated with health-related quality of life. Knowledge of health factors associated to development of a good health-related quality of life could be of use in clinical practice and public health work. The aim of this study was to investigate the associations between suggested health factors and health-related quality of life at baseline and in an eight-year follow up in subjects with and without chronic musculoskeletal pain in a cohort from a general population.

**Methods:**

The study was designed as a longitudinal study in a Swedish general population (N = 1 849) with a postal questionnaire at baseline 1995 and at follow up 2003. Subjects were divided into two groups, according to their response about chronic musculoskeletal pain at baseline. Health-related quality of life was assessed by the SF-36 together with suggested health factors. The associations between SF-36 subscales and suggested health factors were estimated by OR and 95% CI calculated by multivariable logistic regressions, with adjustment for all health factors, age, sex and baseline SF-36 values.

**Results:**

Although subjects without chronic musculoskeletal pain reported better health-related quality of life than subjects with chronic pain, similar health factors were found to be associated to higher scores in SF-36 at baseline and predicted a better outcome in the eight-year follow up. The most consistent finding was a better health outcome in the eight-year follow up for subjects that were feeling rested after sleep. Other factors that in some aspects predicted a better outcome were belonging to higher socioeconomic group, being a native Swede, having emotional support, having good sleep structure, never being or being a former smoker, and regularly drinking alcohol.

**Conclusion:**

The most important health factor in subjects with and without chronic musculoskeletal pain was feeling rested after sleep, but also emotional support, sleep structure, smoking and alcoholic habits appears to be important components. These health factors could be important to address in clinical work with painful musculoskeletal disorders. Since several health factors are common in both subjects with and without pain there could be a common strategy to be formed in public health programmes.

## Background

There is an interest in health care and public health work to identify different risk factors related to disease or ill health in order to optimise prevention and early detection of health problems and poor quality of life [1]. Knowledge of risk factors as well as changes of attitude and life style in the population is supposed to prevent or reduce the burden of disease [2,3]. There has been a focus on prevention and identification of disorders with high mortality, which misses the burden of common diseases, such as musculoskeletal disorders [4,5]. Also in studies of musculoskeletal disorders and health-related quality of life [6,7], the priority has primarily been on risk factors (pathogenesis) instead of the patients' own capacity to adopt factors that promote their health (salutogenesis) [2,3]. Studies primarily looking at risk factors conclude that health could be promoted by several factors, like having a good social network and support, and good work environment [8]. Physical activity is also important [9,10], together with having a good diet [10], normal-weight [11] and being satisfied with sleep [12]. It is also important to reduce the use of alcohol [13] and tobacco [14,15].

Musculoskeletal disorders are major causes to morbidity in the world, and these conditions have a strong negative influence in terms of health-related quality of life [16]. In Europe chronic musculoskeletal pain of moderate or severe intensity occurs in 19% of the adults and these conditions limits the daily activities to a high degree [17]. Musculoskeletal pain is a public health problem and a common cause for people to seek for health care [18-20]. People with musculoskeletal pain, seeking for medical help, estimate their quality of life lower than those who do not seek for medical help [5]. It has also been shown that people with musculoskeletal pain estimates their health-related quality of life very low compared to a pain free population, and that the perceived health can predict musculoskeletal pain outcome [6,7,21].

In order to early identify and reduce the impact on health-related quality of life from musculoskeletal disorders, there is a need for more knowledge regarding factors associated to a good outcome in health. This is of interest both when meeting the patient in the clinical situation and in the public health work aiming to reduce the impact of musculoskeletal disorders in the population. Many factors are proposed to be associated with the development of health-related quality of life [8-15]. It is, from both health promotional and clinical views, of interest to study if there are different patterns of health factors in subjects with and without a chronic condition, such as chronic musculoskeletal pain. Knowledge of factors predicting good health outcome could be used to optimise treatment strategies and health factors common for subjects with and without a musculoskeletal pain could be put forward in a more general health promotion programme. There is a lack of longitudinal studies on health-related quality of life in subjects with or without chronic musculoskeletal pain, focusing on factors that predict health-related quality of life instead of risk factors. The aim of this study was to investigate the associations between suggested health factors and health-related quality of life at baseline and in an eight-year follow up in subjects with and without chronic musculoskeletal pain in a cohort from a general population.

## Methods

### Study design

The study was designed as a longitudinal study in a general population with postal surveys at baseline and at an eight-year follow up, and was a part of the Epipain project [22].

### Subjects and data collection

The target population was all 70 704 inhabitants aged 20–74 years in two municipalities on the west coast of Sweden. In 1995 a sample of 3 928 subjects, representative for the target population, was selected from the official computerised population register. There were 2 425 subjects (62%) who, after two postal reminders, gave their written consent to participation and responded to the initial questionnaire, and 2 332 of those who responded were sent the follow up questionnaire in 2003. Ninety-three subjects were either deceased or had moved abroad. Out of the 2 332 eligible subjects, and after two postal reminders, there were 1 849 respondents (79%) at the follow up.

The 1 849 subjects were divided into two groups, according to their response about chronic musculoskeletal pain. At baseline there were 1 109 (60%) subjects without chronic pain and 700 (38%) subjects with chronic pain. There were missing data about pain for 40 (2%) subjects.

### The Epipain questionnaire

The first part of the questionnaire used in the postal surveys consisted of the well-established Short Form-36 Health Survey (SF-36) in its Swedish standard version [23]. The Swedish version of SF-36 has been found to be reliable and valid [24-26]. The SF-36 is a 36 item questionnaire that gives eight subscales assessing different aspects of health-related quality of life: Physical Functioning (PF), Role – Physical (RP), Bodily Pain (BP), General Health (GH), Vitality (VT), Social Functioning (SF), Role – Emotional (RE) and Mental Health (MH). The score for each of the eight subscales range from 0–100. A higher score indicates better health in that aspect [23].

In the second part of the Epipain questionnaire [22], a majority of the questions were taken from prior studies, where the questions had been found useful. The face and content validity have been found to be good in the second part of the Epipain questionnaire. The reliability was tested with a test-retest and the kappa-value was found to be ranged from 0.8 to 1.0 on the individual questions [27]. The second part of the questionnaire had an overall key question on chronic musculoskeletal pain experience: *Have you experienced pain lasting more than three months during the last twelve months? *It was explained in an introduction that the pain should be persistent or regularly recurrent in the musculoskeletal system. The questionnaire also assessed factors that have been proposed by previous studies to influence the effect on health-related quality of life, such as socioeconomic status, being immigrant, emotional support, regularly exercise, quality of sleep, smoking and alcohol habits [8-10,13-15,22].*Socioeconomic status *was based on an open question regarding the subject's occupation. *Immigrant status *was based on a question regarding if the subjects themselves or at least one of their parents were born in another country. *Emotional support *was based on a question regarding if the subjects have one or more persons who support them to cope with distress and problems in life.*Regularly exercise *was based on a question regarding if the subjects exercise regularly during the week [22]. Quality of *sleep *was based on four questions regarding different aspects of sleep disturbances [22,28,29]. *Smoking habits *were based on a question regarding if the subjects were smokers or not. *Alcohol habits *were based on a question regarding how often the subjects were drinking alcohol [22].

### Statistical procedure and analyses

The subjects were divided into two groups, according to their response about chronic musculoskeletal pain at baseline. Subjects that could not be classified where excluded from the analyses. *Socioeconomic status *was based on the subject's occupation, and classified according to the Swedish socioeconomic classification system, SEI [30]. The 18 basic socioeconomic classes were merged to four groups: manual workers, assistant no manual employees, intermediate/higher no manual employees including upper level executives, and others. The group "others" included self-employed, farmers, housewives, and students [22]. The question regarding *emotional support *could be answered with four alternatives; (1) Yes, definitely, (2) Yes, probably, (3) Not sure, and (4) No. The alternatives 1–2 were dichotomised into Yes and 3–4 into No. The quality of *sleep *was assessed by four questions. Three of these questions were about falling asleep, frequent awakenings during the night and early morning awakening. These questions were regarded to assess the structure or pattern of sleep, and were merged into one group, *sleep structur*e, in the analyses. The worst score in any of the three questions was regarded as representative for problems with sleep structure. The fourth question, not feeling rested after sleep, was regarded to represents a more qualitative aspect of non-restorative sleep and was introduced separately in the analyses as *feeling rested*. Sleep problems were assessed with five alternatives; (1) No problems, (2) Small problems, (3) Some problems, (4) Great problems, and (5) Very great problems. In the analyses the answers were merged into two groups with scores 1–2 representing no/small problems, and scores 3–5 representing moderate/major problems. *Alcohol habits *assessed the frequency of alcohol use with five alternatives; (1) Never, (2) Very seldom, (3) Monthly, (4) 1 or 2 times a week, and (5) Daily. These were merged into three categories with scores 1–2 representing never/rare, 3 representing monthly, and 4–5 representing weekly.

The statistical analyses were done with the statistical package SPSS for Windows, release 15.0. T-test was used for statistical comparison of means. Chi-square-test was used for comparisons of prevalence between groups. The associations between the dependent variables (SF-36 subscales) and independent variables (i.e. the suggested health factors; socioeconomic status, immigrant status, emotional support, regularly exercise, sleep structure, feeling rested, smoking and alcohol habits) were estimated by odds ratios and their 95% confidence intervals calculated by multivariable logistic regressions, with adjustment for all health factors, age, sex and baseline SF-36 values. The SF-36 scores were dichotomised with regard to the mean values in the population for each subscale (1 ≥ mean and 0 < mean). The analyses were done with simple contrast to a reference group for each of the independent variables. At baseline the analyses were checked for interaction between sex or age and all of the independent variables. Subjects with missing values for any of the variables were rejected from the analyses. The actual number of subjects in each analysis is reported in Additional files 1 and 2 (Tables 1–4), and was considered to fulfil the requirement of at least 10 subjects in the outcome for each independent variable. A *P*-value of less than 0.05 was considered statistically significant.

### Ethics

The study was approved by the Ethics Research Committee, Faculty of Medicine, Lund University, Sweden. The Swedish Data Inspection Board approved the computerised registration.

## Results

There was a predominance of women (61% women vs. 39% men; *P *< 0.001) at baseline in subjects with chronic musculoskeletal pain, and a small statistically significant difference for subjects without chronic pain (52% women vs. 48% men; *P *< 0.001). Subjects with chronic pain were significantly older than those without chronic pain (mean age 50.3 vs. 44.6; *P *< 0.001). Details regarding the distribution of sociodemographic characteristics and suggested health factors with regard to the two groups with and without chronic musculoskeletal pain are found in Additional file 3 (Table 5).

### Health-related quality of life at baseline and at the eight-year follow up

**Figure 1:**
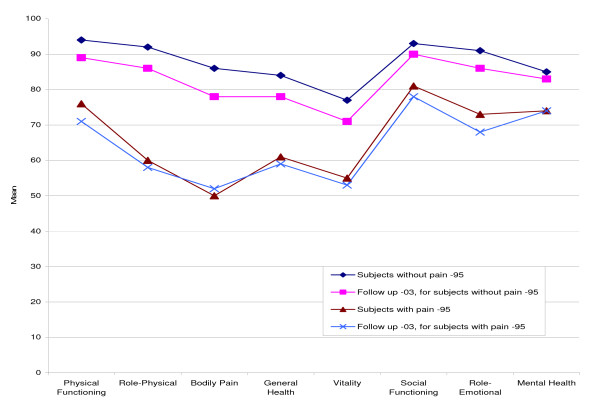
**The SF-36 scores for subjects with and without chronic musculoskeletal pain in 1995 and 2003.** Comparison of the SF-36 subscales scores (mean values) for subjects with and without chronic musculoskeletal pain at baseline in 1995 and at follow up in 2003.

Subjects without chronic musculoskeletal pain scored significantly (*P *< 0.001) better than subjects with chronic musculoskeletal pain in all eight SF-36 dimensions both at baseline and at the eight-year follow up (Figure 1). The scores for all SF-36 dimensions significantly (*P *< 0.001) deteriorated over the eight-year follow up for subjects without chronic pain. The changes were more complex for subjects with chronic pain; significant worsening for PF (*P *< 0.001), SF (*P *= 0.004) and RE (*P *= 0.001), significant improvement for BP (*P *= 0.004), and no significant changes for RP (*P *= 0.368), GH (*P *= 0.419), VT (*P *= 0.391), and MH (*P *= 0.633).

### Factors predicting health-related quality of life at baseline and at the eight-year follow up

The association between suggested health factors and baseline SF-36 mean scores, and the predictive value of these health factors with regard to SF-36 development over eight years, were estimated with multivariable logistic regression analyses, controlling for sociodemographic characteristics. Results from the multivariable logistic regressions with odds ratios (OR) and 95% confidence intervals (CI) for these variables are found in Additional files 1 and 2 (Tables 1–4).

For subjects without chronic musculoskeletal pain at baseline, male *sex *was significantly (*P *< 0.05) associated with having a health status better than the mean score at baseline in PF, VT, SF, RE, and MH, and a worse score in RP. For those with chronic musculoskeletal pain, male sex was significantly associated with having a health status better than the mean score in PF. In the eight-year follow up, male sex significantly predicted a better score only in SF for subjects with chronic pain.

For subjects without chronic musculoskeletal pain at baseline, younger *age *was significantly (*P *< 0.05) associated with having a health status better than the mean score in PF, RP, BP and GH. For those with chronic musculoskeletal pain at baseline, younger age was significantly associated with having a health status better than the mean score for PF, RP, BP and GH. The same pattern could be seen in the eight-year follow up, except that being in the middle age groups significantly predicted better outcome in SF and RE for subjects without chronic pain, and in RE and MH for subjects with chronic pain.

For subjects without chronic musculoskeletal pain at baseline, belonging to a higher *socioeconomic status *was significantly (*P *< 0.05) associated with having a health status better than the mean score in PF. For those with chronic musculoskeletal pain, belonging to a higher socioeconomic status was significantly associated with having a health status better than the mean score in PF, RP, and GH. In the eight-year follow up, a higher socioeconomic status significantly predicted a better outcome in PF for subjects with chronic pain.

For subjects without chronic musculoskeletal pain at baseline, regarding *immigrant status*, being a native Swede was significantly (*P *< 0.05) associated with having a health status better than the mean score in PF, RP, GH, VT, and MH. For those with chronic musculoskeletal pain, being a native Swede was significantly associated with having a health status better than the mean score in GH and VT. In the eight-year follow up, being a native Swede significantly predicted a better outcome in RE and MH both for subjects with and without chronic pain.

For subjects without chronic musculoskeletal pain at baseline, having *emotional support *was significantly (*P *< 0.05) associated with having a health status better than the mean score in RP, GH, VT, SF, RE, and MH. For those with chronic musculoskeletal pain at baseline, emotional support was significantly associated with having a health status better than the mean score in GH, VT, SF, RE, and MH. In the eight-year follow up, emotional support significantly predicted a better outcome in RP, VT, RE, and MH in subjects with chronic pain, but was not significantly predictive in subjects without chronic pain.

For subjects without chronic musculoskeletal pain at baseline, *exercise regularly *was significantly (*P *< 0.05) associated with having a health status better than the mean score in PF, BP, and VT. For those with chronic musculoskeletal pain at baseline, exercise regularly was not significantly associated to any of the eight SF-36 health concepts. In the eight-year follow up exercise regularly failed to significantly predict any outcome in SF-36.

For subjects without chronic musculoskeletal pain at baseline, a good *sleep structure *was significantly (*P *< 0.05) associated with having a health status better than the mean score in all eight SF-36 health concepts except RP. For those with chronic musculoskeletal pain at baseline, a good sleep structure was significantly associated with having a health status better than the mean score in all eight SF-36 health concepts. In the eight-year follow up a good sleep structure significantly predicted a better outcome in PF, RP, and BP for subjects without chronic pain, and in GH and RE for subjects with chronic pain.

For subjects without chronic musculoskeletal pain at baseline, *feeling rested *after sleep was significantly (*P *< 0.05) associated with having a health status better than the mean score in all eight SF-36 health concepts except BP. For those with chronic musculoskeletal pain at baseline, feeling rested was significantly associated with having a health status better than the mean score in all eight SF-36 health concepts except PF. In the eight-year follow up feeling rested significantly predicted a better outcome in BP, GH, VT, SF, RE, and MH for subjects without chronic pain, and in BP, VT, SF, RE, and MH for subjects with chronic pain.

For subjects without chronic musculoskeletal pain at baseline, and regarding *smoking habits*, never being a smoker or being a former smoker, compared to being a current smoker, was significantly (*P *< 0.05) associated with having a health status better than the mean score in PF and MH. For those with chronic musculoskeletal pain at baseline, never being a smoker or being a former smoker, was not significantly associated to any outcome in SF-36. In the eight-year follow up, never being a smoker or being a former smoker significantly predicted a better outcome in GH, SF, and RE for subjects without chronic pain, and in RE and MH for subjects with chronic pain.

For subjects without chronic musculoskeletal pain at baseline, and regarding *alcohol habits*, drinking weekly, compared to never or rarely, was significantly (*P *< 0.05) associated with having a health status better than the mean score in PF, RP, and GH. For those with chronic musculoskeletal pain at baseline, weekly drinking of alcohol was significantly associated with having a health status better than the mean score in BP and VT. In the eight-year follow up weekly drinking of alcohol significantly predicted a better outcome in RP and MH for subjects without chronic pain, and in PF, RP, BP, and RE for subjects with chronic pain.

Although the multivariable logistic regression analyses were not intended to be complete explanatory models, at baseline 8.2–32.7% (Nagelkerke R^2^) of the variance in the dependent variables could be explained by the predictor variables for subjects without chronic pain. The figures were between 16.4–30.9% for subjects with chronic pain. In the eight-year follow up it was 14.5–40.9% for subjects without chronic pain and 26.3–49.5% for subjects with chronic pain.

### Interactions

The analyses where checked for interactions between sex and age, and the suggested health factors at baseline. Although some interactions were noted, few were statistical significant, they were mostly inconsistent and not affecting the main outcome. For subjects with chronic musculoskeletal pain an analysis stratified on sex showed that being a native Swede was associated to better health outcome regarding RP in women but not in men (women: OR 2.95, 95% CI 1.40–6.23; men: OR 0.52, 95% CI 0.20–1.31). The same was noted for RE (women: OR 2.14, 95% CI 1.10–4.15; men: OR 0.52, 95% CI 0.17–1.53). Analyses on subjects with chronic musculoskeletal pain also showed a significant interaction between sex and former smokers in RP (women: OR 2.01, 95% CI 1.05–3.82; men: OR 0.84, 95% CI 0.39–1.82,).

Exercise regularly in age stratified analyses were shown to predict a positive outcome in all eight SF-36 domains for those in the oldest age group but not in the younger age groups for subjects with chronic pain.

## Discussion

Although subjects without chronic musculoskeletal pain reported better health-related quality of life as measured by SF-36 than subjects with chronic musculoskeletal pain, similar health factors were found to be associated to a higher score in health at baseline and also predicted a better outcome in an eight year follow up both in subjects without and with chronic pain. The most consistent finding was a better health outcome for subjects that at baseline were *feeling rested *after sleep. Other factors that in some aspects predicted a better outcome, controlled for age and sex, were belonging to a higher socioeconomic group, being a native Swede, having emotional support, having a good sleep structure, never being or being a former smoker, and regularly drinking alcohol.

Sociodemographic characteristics like being of male sex, younger ages, belonging to a higher socioeconomic status and being a native Swede were associated with having a health status better than the mean score in many of SF-36 health concept at baseline for both subjects with and without chronic musculoskeletal pain. It could be noted that belonging to a younger age group was associated with having a better health status at baseline in the more physical domains of SF-36 (PF, RP, BP, and GH), but not in the more mental (VT, SF, RE, and MH) domains. The same pattern could be seen in the eight year follow up. At the follow up the sociodemographic characteristics, except being of younger age, were of less importance. A study from Texas also showed that being of younger age was important when subjects estimated health but also higher income [10]. The interaction analyses in the present study showed that there could be a sex difference, especially with respect to immigrant status, where being a native Swede was a health factor for women, but not for men. A study from Canada also showed that native-born females reported better health status than foreign-born females [31].

Emotional support was found to be important for a better health-related quality of life at baseline, but at the follow up emotional support was important only for subjects with chronic musculoskeletal pain. Others have reported that emotional support could be very important for the possibilities to handle a disease such as rheumatoid arthritis and its consequences [32]. This strengthens that emotional support could be an important domain to work with in health promoting work.

It was surprising to notice that exercise regularly not was found to be important for health-related quality of life. This is not in accordance with previous studies that have shown a positive association between exercise and health [9,10]. One explanation could be how the term exercise is perceived in different age groups. It is not unlikely that the younger subjects in the present study misunderstood the question and did not count physical activity like walking as exercise. The interaction analyses could be an indicator of this, as physical exercise in age stratified analyses was shown to predict a positive outcome in most SF-36 domains for those in the oldest age group but not in the younger age groups for subjects with chronic pain. Future studies have to take this into consideration and questions may have to be more precise regarding level of physical activity. There could also be complex interactions between exercise and a number of other factors that reduce the impact of exercise in this study, which includes several other health predicting variables in the analyses.

In this study having a good sleep structure was associated to a higher score in health-related quality of life both in subjects with and without chronic musculoskeletal pain at baseline, and also predicted a better outcome over eight years in several SF-36 domains. In another study insufficient sleep also has been associated with impairment of health-related quality of life but also with frequent pain [12]. Our study indicates together with findings in other studies [33,34] that a good sleep structure could be an important domain to work with in health promoting work.

Feeling rested after sleep was the most important of the studied health factors, predicting a better outcome in most of the SF-36 dimensions for both subjects with and without chronic musculoskeletal pain at both baseline and follow up. Earlier studies have shown that tiredness and fatigue are very common symptoms in people with different diseases, for example rheumatoid arthritis and cancer [35,36]. But in this study we also could show that feeling rested was important even in subjects without chronic musculoskeletal pain and how they reported their health-related quality of life. The consistent finding of feeling rested as an important health factor highlight that this has to be taken into account both in care of patients and in all health promoting work.

In this study, especially at the follow up, never being a smoker or being a former smoker, compared to being current smokers, was associated to a better health-related quality of life both in subjects with and without chronic musculoskeletal pain. It has also been reported by others that those who never have smoked or were former smokers have smaller impairment on the health-related quality of life compared with subjects who were current smokers [14]. In Australia, they have found that female smokers estimated their health-related quality of life lower than female non-smokers and men smokers [15]. This is interesting with regard to the findings in this study with interaction analyses on sex, where former smoking was associated to better score in baseline SF-36 domains RP and RE in females, but not in men. This could indicate a sex difference that has to be considered in future studies.

Drinking alcohol weekly was significantly associated with having a better health status for both subjects with and without chronic musculoskeletal pain at baseline and at follow up. One earlier study has shown that people that rate their health status low was drinking alcohol more often than people with good health status [13]. Another study presented that men with frequent sleep insufficiency drink alcohol heavily [12]. In our study the quantity of alcohol was not recorded and any comparable conclusions can not be drawn. Thus, further studies have to be done with more questions about the alcohol habits and its importance for the health-related quality of life.

The factors associated to good health presented in this study could be important to address in clinical work with patients having painful musculoskeletal disorders, in order to enhance the effect of medical treatment for the disease. Since several factors are common in subjects with and without chronic musculoskeletal pain a common strategy could be formed in public health programmes on national and international levels [37].

Since SF-36 is a generic measure of health status the outcome over eight years could be expected to be influenced by a large number of factors, including the development of chronic musculoskeletal pain. In the design of the study it was decided to study two cohorts based on the baseline pain report and not to include the change in pain status in the forming of the groups. The forming of multiple groups, based on pain progression, was considered to give at too complex picture. Changes in pain status could though, together with other concomitant disorders, explain changes in health. Since predictors of pain development have been reported in several studies [6,7], it was decided not to study the progression of musculoskeletal pain, but to focus on health status as outcome in this study.

### Possible confounders, bias and misclassifications

In an analysis of non-responders in a prior work of this population, it was found that people with chronic musculoskeletal pain were more prone to respond than people without musculoskeletal pain, giving a higher estimate of the prevalence [22]. This is not likely to bias the results in the follow up of the cohorts that were established in 1995.

The material was thoroughly checked for errors and subjects that could not be classified to have pain or not, were excluded from the analyses.

In our study we were not searching for the optimal model of health factors predicting health-related quality of life, therefore goodness-of-fit statistics were not tested and reported.

As age and sex were likely to be confounders we controlled for these factors in the analyses. We also controlled the baseline value for every subscale in SF-36 at the follow up to adjust for the possibility that outcome would reflect the baseline score and not a change over time. There is a problem in the use of SF-36 that floor and roof effects can reduce the possible change over time in the extreme ends of the scales.

## Conclusion

The most important health factor for both subjects with and without chronic musculoskeletal pain was the report of feeling rested after sleep, associated both to a better score in most of the SF-36 dimension at baseline and predicting a better outcome at the eight-year follow up. Other health factors predicting better health-related quality of life were having emotional support, having a good sleep structure, never being or being a former smoker and regularly drinking alcohol for both subjects with and without chronic pain. These health promoting factors could be important to address in clinical work with patients having painful musculoskeletal disorders. Since several health factors are common in subjects with and without chronic musculoskeletal pain, there could be a common strategy to be formed in public health programmes on national and international levels.

## Competing interests

The authors declare that they have no competing interests.

## Authors' contributions

All authors contribution equally in designing the study, discussing the statistical framework, interpretation and discussion of the findings. SA and SB carried out the statistical analyses and drafted the manuscript. All authors read and approved the final manuscript.

## Supplementary Material

Additional file 1 **Table 1–2.** Factors believed to affect health-related quality of life in a general population at baseline 1995. Odds ratios (95% CI) in multivariable analyses of factors believed to affect health-related quality of life (assessed by SF-36) in a positive way in a general population with and without chronic musculoskeletal pain at baseline 1995. Click here for file

Additional file 2 **Table 3–4.** Baseline factors believed to affect health-related quality of life in a general population eight years later. Odds ratios (95% CI) in multivariable analyses of baseline factors believed to affect health-related quality of life (assessed by SF-36) in a positive way in a general population with and without chronic musculoskeletal pain eight years later. Click here for file

Additional file 3 **Table 5.** Sociodemographic and supposed health-factors. Sociodemographic and supposed health-factors among a general population with and without chronic pain in 1995. Click here for file
