# Supplementary material for: Health predicting factors in a general population over an eight-year period in subjects with and without chronic musculoskeletal pain
Source: Health Qual Life Outcomes. 2008 Nov 11;6:98. doi: 10.1186/1477-7525-6-98 (PMC2636776; doi:10.1186/1477-7525-6-98)
Supplement: Additional file 2 — Table 3–4. Baseline factors believed to affect health-related quality of life in a general population eight years later. Odds ratios (95% CI) in multivariable analyses of baseline factors believed to affect health-related quality of life (assessed by SF-36) in a positive way in a general population with and without chronic musculoskeletal pain eight years later. [file 1477-7525-6-98-S2.doc]

Additional file 2

Table 3. Baseline factors believed to affect health-related quality of life in a general population eight years later

Odds ratios (95 % CI) in multivariable analyses of baseline factors believed to affect health-related quality of life (assessed by SF-36) in a positive way in a

general population with and without chronic musculoskeletal pain eight years later

|  |  | Physical function (PF) | | Role-Physical (RP) | | Bodily pain (BP) | | General health (GH) | |
| --- | --- | --- | --- | --- | --- | --- | --- | --- | --- |
|  |  | Without pain  n= 1011  OR (95 % CI) | With pain  n= 634  OR (95 % CI) | Without pain  n= 1004  OR (95 % CI) | With pain  n= 615  OR (95 % CI) | Without pain  n= 1029  OR (95 % CI) | With pain  n= 651  OR (95 % CI) | Without pain  n= 1005  OR (95 % CI) | With pain  n= 631  OR (95 % CI) |
| Sex | Women | 1.00 | 1.00 | 1.00 | 1.00 | 1.00 | 1.00 | 1.00 | 1.00 |
|  | Men | 1.3 (0.8-1.9) | 1.2 (0.8-1.4) | 1.2 (0.9-1.7) | 1.1 (0.7-1.6) | 1.1 (0.8-1.4) | 0.8 (0.5-1.3) | 0.8 (0.6-1.1) | 1.1 (0.8-1.7) |
|  |  |  |  |  |  |  |  |  |  |
| Age | 59-74 | 1.00 | 1.00 | 1.00 | 1.00 | 1.00 | 1.00 | 1.00 | 1.00 |
| (years) | 47-58 | 3.6 (2.2-6.1) | 3.4 (1.9-5.9) | 2.9 (1.8-4.6) | 1.4 (0.9-2.3) | 1.5 (0.9-2.3) | 1.7 (0.9-3.0) | 2.2 (1.3-3.5) | 1.5 (0.9-2.5) |
|  | 34-46 | 6.3 (3.5-11.2) | 6.5 (3.4-12.1) | 3.1 (1.9-5.0) | 3.0 (1.8-5.2) | 1.6 (1.1-2.5) | 3.1 (1.7-5.7) | 1.6 (0.9-2.5) | 2.5 (1.4-4.3) |
|  | 20-33 | 10.3 (5.4-19.4) | 9.4 (4.6-19.1) | 3.6 (2.2-5.8) | 2.2 (1.2-3.9) | 1.9 (1.2-2.9) | 3.3 (1.7-6.3) | 3.4 (2.1-5.6) | 2.5 (1.3-4.6) |
|  |  |  |  |  |  |  |  |  |  |
| Socio- | Group A | 1.00 | 1.00 | 1.00 | 1.00 | 1.00 | 1.00 | 1.00 | 1.00 |
| economic | Group B | 1.1 (0.6-2.0) | 1.1 (0.6-2.0) | 0.8 (0.5-1.3) | 0.7 (0.4-1.3) | 1.0 (0.7-1.6) | 1.0 (0.5-1.8) | 0.7 (0.5-1.2) | 0.9 (0.5-1.6) |
| status | Group C | 1.4 (0.9-2.3) | 2.0 (1.2-3.5) | 1.3 (0.9-2.0) | 1.0 (0.6-1.7) | 1.4 (0.9-2.0) | 1.0 (0.6-1.7) | 1.2 (0.8-1.8) | 1.2 (0.7-2.0) |
|  | Group D | 0.8 (0.4-1.6) | 1.2 (0.6-2.6) | 0.8 (0.5-1.3) | 0.9 (0.5-1.7) | 0.9 (0.5-1.4) | 0.9 (0.4-1.8) | 0.6 (0.3-0.9) | 1.0 (0.5-2.0) |
|  |  |  |  |  |  |  |  |  |  |
| Immigrant | Immigrant | 1.00 | 1.00 | 1.00 | 1.00 | 1.00 | 1.00 | 1.00 | 1.00 |
| status | Swede | 1.5 (0.7-2.9) | 1.2 (0.6-2.3) | 1.4 (0.8-2.4) | 1.5 (0.8-2.7) | 1.1 (0.6-1.8) | 0.8 (0.4-1.4) | 1.5 (0.8-2.5) | 1.5 (0.8-2.9) |
|  |  |  |  |  |  |  |  |  |  |
| Emotional | No | 1.00 | 1.00 | 1.00 | 1.00 | 1.00 | 1.00 | 1.00 | 1.00 |
| support | Yes | 0.8 (0.4-1.5) | 1.0 (0.5-1.7) | 1.6 (0.9-2.6) | 1.7 (1.0-2.8) | 1.3 (0.8-2.1) | 1.8 (0.9-3.5) | 1.0 (0.6-1.7) | 1.2 (0.7-2.1) |
|  |  |  |  |  |  |  |  |  |  |
| Exercise | No | 1.00 | 1.00 | 1.00 | 1.00 | 1.00 | 1.00 | 1.00 | 1.00 |
| regularly | Yes, 1-2 times  a week | 1.0 (0.6-1.6) | 0.9 (0.6-1.5) | 1.1 (0.7-1.5) | 1.1 (0.7-1.7) | 0.9 (0.6-1.2) | 0.9 (0.5-1.4) | 1.4 (0.9-2.0) | 0.9 (0.6-1.4) |
|  | Yes, > 2 times  a week | 1.0 (0.6-1.7) | 0.7 (0.4-1.2) | 0.9 (0.6-1.4) | 0.9 (0.6-1.5) | 1.1 (0.8-1.7) | 1.6 (0.9-2.7) | 1.4 (0.9-2.2) | 1.1 (0.7-1.8) |
|  |  |  |  |  |  |  |  |  |  |
| Sleep | Bad | 1.00 | 1.00 | 1.00 | 1.00 | 1.00 | 1.00 | 1.00 | 1.00 |
| structure | Good | 1.7 (1.0-2.6) | 0.7 (0.4-1.2) | 1.7 (1.2-2.5) | 1.5 (0.9-2.3) | 1.4 (1.0-2.0) | 1.1 (0.7-1.7) | 1.2 (0.8-1.7) | 1.8 (1.1-2.8) |
|  |  |  |  |  |  |  |  |  |  |
| Feeling | No | 1.00 | 1.00 | 1.00 | 1.00 | 1.00 | 1.00 | 1.00 | 1.00 |
| rested | Yes | 1.2 (0.7-2.1) | 1.5 (0.9-2.4) | 1.3 (0.9-2.1) | 1.4 (0.9-2.1) | 1.9 (1.3-2.9) | 2.2 (1.4-3.6) | 1.6 (1.0-2.5) | 1.2 (0.8-1.9) |
|  |  |  |  |  |  |  |  |  |  |
| Smoking | Current | 1.00 | 1.00 | 1.00 | 1.00 | 1.00 | 1.00 | 1.00 | 1.00 |
| habit | Former | 1.3 (0.7-2.4) | 0.9 (0.5-1.6) | 1.5 (0.9-2.4) | 0.8 (0.5-1.4) | 1.4 (0.9-2.2) | 1.4 (0.8-2.5) | 2.2 (1.3-3.5) | 1.3 (0.8-2.3) |
|  | Never | 1.1 (0.6-1.8) | 1.5 (0.9-2.6) | 1.1 (0.7-1.6) | 1.3 (0.8-2.2) | 1.2 (0.8-1.7) | 1.7 (0.9-3.0) | 1.5 (1.0-2.3) | 1.3 (0.8-2.2) |
|  |  |  |  |  |  |  |  |  |  |
| Alcohol | Never/rare | 1.00 | 1.00 | 1.00 | 1.00 | 1.00 | 1.00 | 1.00 | 1.00 |
| habit | Monthly | 1.3 (0.8-2.0) | 1.4 (0.9-2.3) | 1.3 (0.9-1.8) | 1.8 (1.1-2.7) | 1.3 (0.9-1.9) | 1.9 (1.1-3.1) | 1.3 (0.9-1.9) | 1.4 (0.9-2.2) |
|  | Weekly | 1.6 (0.9-2.9) | 2.7 (1.5-4.8) | 1.7 (1.0-2.7) | 2.1 (1.2-3.5) | 1.3 (0.9-2.0) | 2.0 (1.1-3.6) | 1.3 (0.8-2.1) | 1.3 (0.7-2.3) |
|  |  |  |  |  |  |  |  |  |  |
| SF-36 at | >mean | 1.00 | 1.00 | 1.00 | 1.00 | 1.00 | 1.00 | 1.00 | 1.00 |
| baseline | <mean | 8.8 (5.5-14.2) | 9.1 (5.8-14.3) | 2.8 (1.9-4.3) | 3.8 (2.6-5.5) | 4.6 (3.2-6.6) | 5.7 (3.6-9.2) | 5.7 (4.0-8.2) | 6.9 (4.5-10.4) |

Group A: Manual workers

Group B: Assistant no manual employees

Group C: Intermediate/higher employees and upper-level executives

Group D: Others

Table 4. Baseline factors believed to affect health-related quality of life in a general population eight years later

Odds ratios (95 % CI) in multivariable analyses of baseline factors believed to affect health-related quality of life (assessed by SF-36) in a positive way in a

general population with and without chronic musculoskeletal pain eight years later

|  |  | Vitality (VT) | | Social Functioning (SF) | | Role-Emotional (RE) | | Mental Health (MH) | |
| --- | --- | --- | --- | --- | --- | --- | --- | --- | --- |
|  |  | Without pain  n= 1017  OR (95 % CI) | With pain  n= 639  OR (95 % CI) | Without pain  n= 1029  OR (95 % CI) | With pain  n= 652  OR (95 % CI) | Without pain  n= 999  OR (95 % CI) | With pain  n= 619  OR (95 % CI) | Without pain  n= 1018  OR (95 % CI) | With pain  n= 638  OR (95 % CI) |
| Sex | Women | 1.00 | 1.00 | 1.00 | 1.00 | 1.00 | 1.00 | 1.00 | 1.00 |
|  | Men | 1.2 (0.8-1.6) | 1.4 (0.9-2.0) | 1.4 (0.9-1.9) | 1.5 (1.0-2.2) | 1.4 (0.9-1.9) | 1.1 (0.7-1.6) | 1.2 (0.9-1.6) | 1.4 (0.9-2.1) |
|  |  |  |  |  |  |  |  |  |  |
| Age | 59-74 | 1.00 | 1.00 | 1.00 | 1.00 | 1.00 | 1.00 | 1.00 | 1.00 |
| (years) | 47-58 | 1.6 (0.9-2.5) | 1.1 (0.7-1.7) | 1.8 (1.1-2.9) | 1.0 (0.6-1.6) | 1.7 (1.0-2.7) | 1.3 (0.8-2.1) | 0.9 (0.5-1.5) | 2.1 (1.3-3.3) |
|  | 34-46 | 1.1 (0.7-1.7) | 1.6 (0.9-2.7) | 1.3 (0.8-2.1) | 1.6 (0.9-2.8) | 2.1 (1.3-3.4) | 2.3 (1.4-3.9) | 0.7 (0.4-1.2) | 1.7 (1.0-2.9) |
|  | 20-33 | 1.0 (0.6-1.6) | 1.2 (0.7-2.1) | 1.2 (0.7-1.9) | 0.9 (0.5-1.6) | 1.6 (0.9-2.5) | 1.6 (0.9-2.8) | 0.7 (0.4-1.1) | 0.8 (0.4-1.4) |
|  |  |  |  |  |  |  |  |  |  |
| Socio- | Group A | 1.00 | 1.00 | 1.00 | 1.00 | 1.00 | 1.00 | 1.00 | 1.00 |
| economic | Group B | 1.0 (0.6-1.7) | 1.0 (0.6-1.7) | 0.6 (0.4-0.9) | 1.2 (0.7-2.1) | 0.7 (0.4-1.1) | 1.1 (0.7-1.9) | 0.9 (0.6-1.5) | 1.0 (0.6-1.7) |
| status | Group C | 1.3 (0.9-1.8) | 0.9 (0.6-1.5) | 0.9 (0.6-1.3) | 0.8 (0.5-1.4) | 0.8 (0.5-1.1) | 1.0 (0.6-1.6) | 1.1 (0.8-1.6) | 0.9 (0.6-1.5) |
|  | Group D | 0.8 (0.5-1.3) | 0.8 (0.4-1.4) | 0.6 (0.4-1.0) | 1.1 (0.6-2.1) | 1.0 (0.6-1.7) | 0.8 (0.4-1.5) | 0.9 (0.6-1.5) | 1.2 (0.6-2.2) |
|  |  |  |  |  |  |  |  |  |  |
| Immigrant | Immigrant | 1.00 | 1.00 | 1.00 | 1.00 | 1.00 | 1.00 | 1.00 | 1.00 |
| status | Swede | 1.4 (0.9-2.4) | 1.2 (0.7-2.3) | 1.6 (0.9-2.6) | 1.4 (0.8-2.4) | 1.8 (1.1-3.1) | 1.8 (1.0-3.3) | 1.9 (1.1-3.1) | 1.9 (1.1-3.4) |
|  |  |  |  |  |  |  |  |  |  |
| Emotional | No | 1.00 | 1.00 | 1.00 | 1.00 | 1.00 | 1.00 | 1.00 | 1.00 |
| support | Yes | 1.3 (0.8-2.1) | 1.7 (1.0-3.0) | 1.6 (0.9-2.6) | 1.4 (0.8-2.2) | 1.6 (0.9-2.7) | 1.7 (1.1-2.8) | 1.5 (0.9-2.5) | 2.0 (1.2-3.4) |
|  |  |  |  |  |  |  |  |  |  |
| Exercise | No | 1.00 | 1.00 | 1.00 | 1.00 | 1.00 | 1.00 | 1.00 | 1.00 |
| regularly | Yes, 1-2 times  a week | 1.0 (0.7-1.5) | 1.1 (0.7-1.7) | 1.0 (0.7-1.4) | 1.0 (0.7-1.5) | 1.1 (0.8-1.7) | 1.0 (0.7-1.6) | 1.2 (0.9-1.7) | 0.7 (0.5-1.1) |
|  | Yes, > 2 times  a week | 0.8 (0.5-1.1) | 1.1 (0.7-1.7) | 0.7 (0.5-1.1) | 1.0 (0.6-1.6) | 1.1 (0.7-1.7) | 1.1 (0.7-1.8) | 1.0 (0.7-1.5) | 1.3 (0.8-2.0) |
|  |  |  |  |  |  |  |  |  |  |
| Sleep | Bad | 1.00 | 1.00 | 1.00 | 1.00 | 1.00 | 1.00 | 1.00 | 1.00 |
| structure | Good | 1.3 (0.9-1.8) | 1.3 (0.8-2.0) | 1.3 (0.9-1.9) | 1.4 (0.9-2.2) | 1.2 (0.8-1.8) | 1.6 (1.0-2.5) | 1.0 (0.7-1.5) | 1.5 (0.9-2.4) |
|  |  |  |  |  |  |  |  |  |  |
| Feeling | No | 1.00 | 1.00 | 1.00 | 1.00 | 1.00 | 1.00 | 1.00 | 1.00 |
| rested | Yes | 2.1 (1.4-3.2) | 1.6 (1.1-2.5) | 1.7 (1.1-2.7) | 1.6 (1.0-2.4) | 1.8 (1.2-2.7) | 1.6 (1.0-2.4) | 2.7 (1.8-4.1) | 1.6 (1.1-2.4) |
|  |  |  |  |  |  |  |  |  |  |
| Smoking | Current | 1.00 | 1.00 | 1.00 | 1.00 | 1.00 | 1.00 | 1.00 | 1.00 |
| habit | Former | 1.4 (0.9-2.2) | 1.1 (0.7-1.9) | 2.1 (1.3-3.3) | 1.0 (0.6-1.7) | 1.7 (1.0-2.8) | 1.4 (0.8-2.3) | 1.5 (0.9-2.4) | 1.6 (0.9-2.7) |
|  | Never | 1.3 (0.9-1.9) | 1.1 (0.7-1.7) | 1.7 (1.2-2.6) | 1.5 (0.9-2.4) | 1.3 (0.8-1.9) | 1.9 (1.2-3.1) | 1.3 (0.9-1.9) | 1.6 (1.0-2.6) |
|  |  |  |  |  |  |  |  |  |  |
| Alcohol | Never/rare | 1.00 | 1.00 | 1.00 | 1.00 | 1.00 | 1.00 | 1.00 | 1.00 |
| habit | Monthly | 1.1 (0.7-1.5) | 1.1 (0.7-1.7) | 1.0 (0.7-1.4) | 1.3 (0.8-1.9) | 1.4 (0.9-2.0) | 1.5 (0.9-2.3) | 1.5 (1.0-2.1) | 0.9 (0.6-1.4) |
|  | Weekly | 1.2 (0.8-1.9) | 1.4 (0.8-2.3) | 1.1 (0.7-1.8) | 1.2 (0.7-1.9) | 1.2 (0.8-1.9) | 2.0 (1.2-3.4) | 1.1 (0.7-1.8) | 1.1 (0.7-1.9) |
|  |  |  |  |  |  |  |  |  |  |
| SF-36 at | >mean | 1.00 | 1.00 | 1.00 | 1.00 | 1.00 | 1.00 | 1.00 | 1.00 |
| baseline | <mean | 4.2 (3.0-5.9) | 4.3 (2.9-6.3) | 3.0 (2.1-4.4) | 4.7 (3.2-6.8) | 2.9 (2.0-4.4) | 3.2 (2.2-4.7) | 3.0 (2.1-4.2) | 4.1 (2.8-5.9) |

Group A: Manual workers

Group B: Assistant no manual employees

Group C: Intermediate/higher employees and upper-level executives

Group D: Others
