# Supplementary material for: Health predicting factors in a general population over an eight-year period in subjects with and without chronic musculoskeletal pain
Source: Health Qual Life Outcomes. 2008 Nov 11;6:98. doi: 10.1186/1477-7525-6-98 (PMC2636776; doi:10.1186/1477-7525-6-98)
Supplement: Additional file 3 — Table 5. Sociodemographic and supposed health-factors. Sociodemographic and supposed health-factors among a general population with and without chronic pain in 1995. [file 1477-7525-6-98-S3.doc]

Additional file 3

Table 5. Sociodemographic and supposed health-factors among

a general population with and without chronic pain in 1995

* Statistically significant (p<0.05) difference between the two groups

| Characteristics | Without pain | |  | With pain | |
| --- | --- | --- | --- | --- | --- |
|  | n=1109 | % |  | n=700 | % |
| *Sex  Women  Men | 578  531 | 52.1  47.9 |  | 429  271 | 61.3  38.7 |
|  |  |  |  |  |  |
| *Age (years)  20-33  34-46  47-58  59-74 | 303  295  291  220 | 27.3  26.6  26.2  19.8 |  | 107  155  215  223 | 15.3  22.1  30.7  31.9 |
|  |  |  |  |  |  |
| *Socioeconomic status  Manual workers  Assistant no manual employees  Intermediate/higher employees and  upper-level executives  Others | 463  150  348  148 | 41.7  13.5  31.4  13.3 |  | 386  99  139  76 | 55.1  14.1  19.9  10.9 |
|  |  |  |  |  |  |
| *Immigrant status  Immigrant  Swede | 94  1009 | 8.5  91.5 |  | 90  604 | 13.0  87.0 |
|  |  |  |  |  |  |
| *Emotional support  No  Yes | 107  995 | 9.7  90.3 |  | 127  570 | 18.2  81.8 |
|  |  |  |  |  |  |
| Exercise regularly  No  Yes, 1-2 times a week  Yes, >2 times a week | 448  388  261 | 40.8  35.4  23.8 |  | 304  230  159 | 43.9  33.2  22.9 |
|  |  |  |  |  |  |
| *Sleep structure  Bad  Good | 321  763 | 29.6  70.4 |  | 416  269 | 60.7  39.3 |
|  |  |  |  |  |  |
| *Feeling rested  No  Yes | 189  877 | 17.7  82.3 |  | 347  323 | 51.8  48.2 |
|  |  |  |  |  |  |
| *Smoking habit  Current  Former | 217  270 | 19.7  24.5 |  | 159  208 | 22.8  29.8 |
| Never | 616 | 55.8 |  | 331 | 47.4 |
|  |  |  |  |  |  |
| *Alcohol habit  Never/rare  Monthly | 331  493 | 30.0  44.6 |  | 292  252 | 42.0  36.3 |
| Weekly | 281 | 25.4 |  | 151 | 21.7 |
